# ExoHCR: a sensitive assay to profile PD-L1 level on tumor exosomes for immunotherapeutic prognosis

**DOI:** 10.1007/s41048-020-00122-x

**Published:** 2020-11-23

**Authors:** Lujun Hu, Wenjie Chen, Shurong Zhou, Guizhi Zhu

**Affiliations:** 1 College of Bioengineering, Sichuan University of Science and Engineering, Zigong 643000, Sichuan, China; 2 Center for Pharmaceutical Engineering and Sciences, Department of Pharmaceutics, School of Pharmacy, Virginia Commonwealth University, Richmond, VA 23298, USA; 3 The Developmental Therapeutics Program, Massey Cancer Center, Virginia Commonwealth University, Richmond, VA 23298, USA; 4 Institute for Structural Biology, Drug Discovery and Development, Virginia Commonwealth University, Richmond, VA 23219, USA

**Keywords:** Exosome, Hybridization chain reaction, Immune checkpoint, PD-L1 analysis, Immunotherapy

## Abstract

Cancer immunotherapy has made recent breakthrough, including immune checkpoint blockade (ICB) that inhibits immunosuppressive checkpoints such as programmed cell death protein 1 (PD-1) and programmed death-ligand 1 (PD-L1). However, most cancer patients do not durably respond to ICB. To predict ICB responses for patient stratification, conventional immunostaining has been used to analyze the PD-L1 expression level on biopsied tumor tissues but has limitations of invasiveness and tumor heterogeneity. Recently, PD-L1 levels on tumor cell exosomes showed the potential to predict ICB response. Here, we developed a non-invasive, sensitive, and fast assay, termed as exosome-hybridization chain reaction (ExoHCR), to analyze tumor cell exosomal PD-L1 levels. First, using αCD63-conjugated magnetic beads, we isolated exosomes from B16F10 melanoma and CT26 colorectal cancer cells that were immunostimulated to generate PD-L1-positive exosomes. Exosomes were then incubated with a conjugate of PD-L1 antibody with an HCR trigger DNA (T), in which one αPD-L1-T conjugate carried multiple copies of T. Next, a pair of metastable fluorophore-labeled hairpin DNA (H1 and H2) were added, allowing T on αPD-L1-T to initiate HCR *in situ* on bead-conjugated exosome surfaces. By flow cytometric analysis of the resulting beads, relative to αPD-L1-fluorophore conjugates, ExoHCR amplified the fluorescence signal intensities for exosome detection by 3–7 times in B16F10 cells and CT26 cells. Moreover, we validated the biostability of ExoHCR in culture medium supplemented with 50% FBS. These results suggest the potential of ExoHCR for non-invasive, sensitive, and fast PD-L1 exosomal profiling in patient stratification of cancer immunotherapy.

## INTRODUCTION

Cancer immunotherapy has made significant progress over the past decade (Dougan and Dranoff 2009; Khalil *et al*. 2016). ICB has been one of the most successful approaches to cancer immunotherapy thus far. ICB agents have been developed for a variety of immune checkpoints such as PD-1, PD-L1, and cytotoxic T-lymphocyte-associated protein 4 (CTLA-4) (Ribas and Wolchok 2018). However, overall, only a small subset of cancer patients can respond to current ICB agents (Farkona *et al*. 2016). This challenge is aggravated by the fact that the therapeutic responses of cancer patients to ICB cannot be determined until weeks later, by when the non-responding patients may have missed the best opportunities for other treatment. These challenges call for rapid and sensitive prognostics that predict the therapeutic responses of patients to ICB in patient stratification. PD-L1 expression levels can aid the prediction of the cancer therapeutic efficacy of multiple immune checkpoint inhibitors such as αPD-1 and αPD-L1 (Chen *et al*. 2018; Poggio *et al*. 2019). In a current clinical setting, PD-L1 levels are determined by immunohistochemistry (IHC)-based assays using tumor tissues. However, IHC-based assays have drawbacks such as invasiveness, and poor sensitivity (Shia 2008). To address these challenges, fast, sensitive, and noninvasive prognostics that profile PD-L1 expression levels would be highly desired to predict ICB therapeutic responses.

Recent reports showed that the PD-L1 expression levels on tumor exosomes are correlated to the PD-L1 levels on the corresponding tumor cells. More importantly, the tumor exosome PD-L1 levels are correlated with the responsiveness of tumors to the corresponding ICB (Haderk *et al*. 2017). Exosomes are cell-derived nanovesicles with a size of 30–120 nm which are present in many bodily fluids, including blood, urine, saliva, amniotic fluid, breast milk, hydrothoracic fluid, and ascitic fluid, as well as in culture medium of most cell types under both physiological and pathological conditions, especially tumor cells (Théry *et al*. 2002). The involvement of exosomes in immunity suggests that they have enormous potential for human pathology as reservoirs of diagnostic and prognostic biomarkers (De Toro *et al*. 2015). Derived from cells, exosomes may represent their parental cells in non-invasive monitoring of various biological processes, including immunooncological pathways (Xu *et al*. 2018). Besides, compared to tumor biopsies, exosomes, which can be accessed by traditional minimally invasive liquid biopsy, can accurately reflect tumor status in real time and bypass the limitations of tumor heterogeneity and sampling bias (Azmi *et al*. 2013). Taken together, PD-L1 levels of exosomes hold great potential to be a prognostic biomarker for ICB-related patient stratification (Song *et al*. 2019).

HCR is an enzyme-free DNA reaction which has been employed for isothermal signal amplification in bio-analysis (Dirks and Pierce 2004; Zhu *et al*. 2013a, b; Choi *et al*. 2014). Briefly, upon initiation by a trigger DNA, HCR self-assembles trigger-DNA-tethered long double-stranded DNA (dsDNA) chains from short hairpin monomers via a cascade of DNA hybridization (Dirks and Pierce 2004). Signal amplification based on HCR is enzyme-free, isothermal, and rapid, in contrast to complex thermocycling used by enzyme-catalyzed polymerase chain reaction (PCR). As such, HCR has been extensively studied for application in bioanalysis, point-of-care diagnosis and prognosis, as well as biotechnology (Wu *et al*. 2015; Huang *et al*. 2011; Xuan and Hsing 2014; Ren *et al*. 2011).

**Fig. 1:**
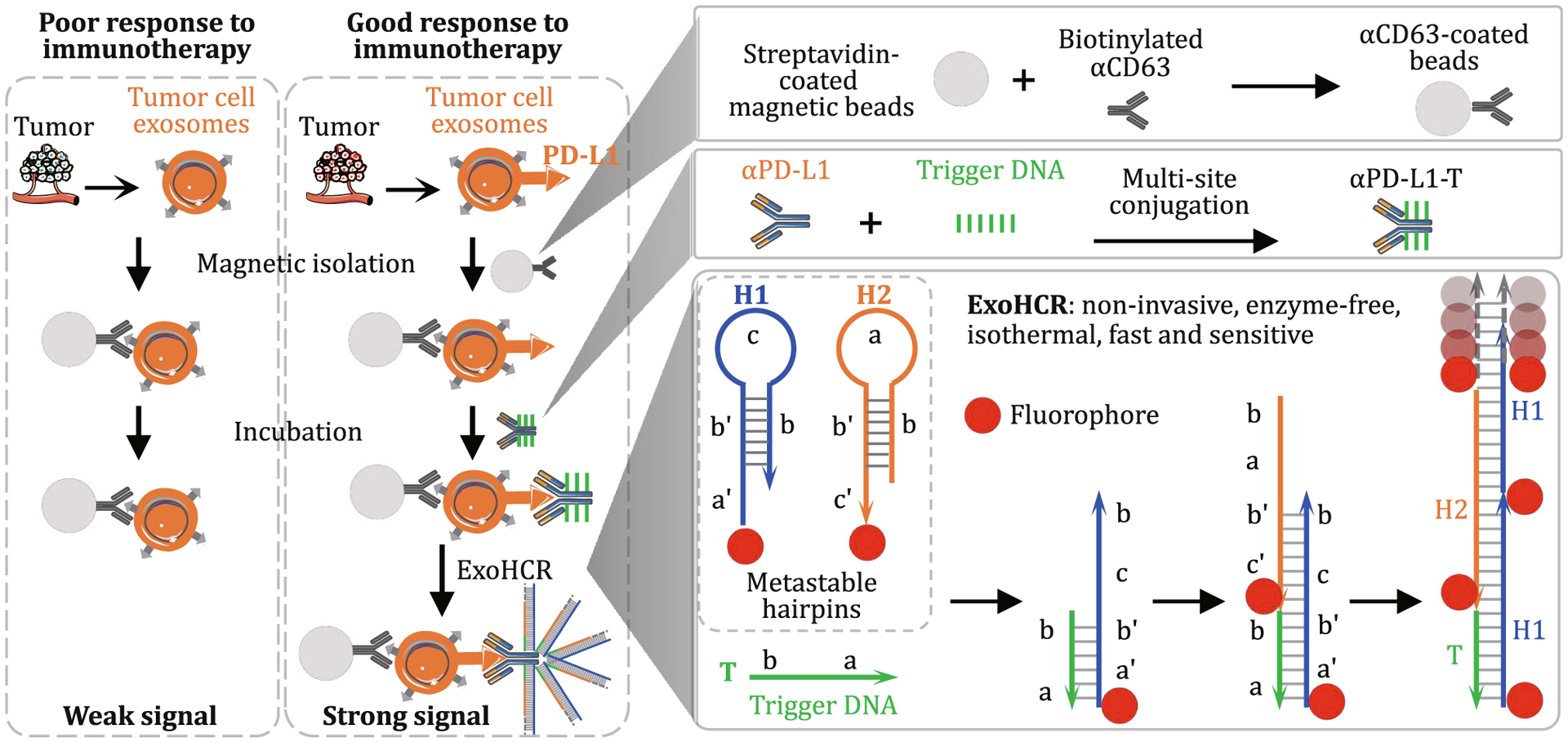
Schematic illustration of ExoHCR to profile PD-L1 levels on tumor cell exosomes for potential application in the predication of responsiveness to cancer immunotherapy

Here, we developed a non-invasive, isothermal, sensitive, and fast assay, termed as exosome-hybridization chain reaction (ExoHCR) for PD-L1 level profiling on tumor cell exosomes, with the long-term goal of ICB-related therapeutic response prediction and patient stratification (Fig. 1). Specifically, multiple copies of trigger DNA (T) were linked with one αPD-L1. The resulting αPD-L1-T was incubated with tumor cell exosomes that were harvested using magnetic beads from immune-stimulated tumor cells. After washing off unbound αPD-L1-T, dye-labeled H1 and H2 were added, resulting in the initiation of HCR and the formation of long dsDNA on αPD-L1, which was bound with exosomes. As a result, ExoHCR significantly amplified the fluorescence signal intensities on exosomes in only 1.5 h. These results indicate the potential of ExoHCR as a non-invasive, isothermal, sensitive, and fast assay for PD-L1 level profiling on tumor cell exosomes, in order for ICB-related therapeutic response prediction and patient stratification.

## RESULTS AND DISCUSSION

### Generation and isolation of tumor cell-derived exosomes with surface PD-L1

**Fig. 2:**
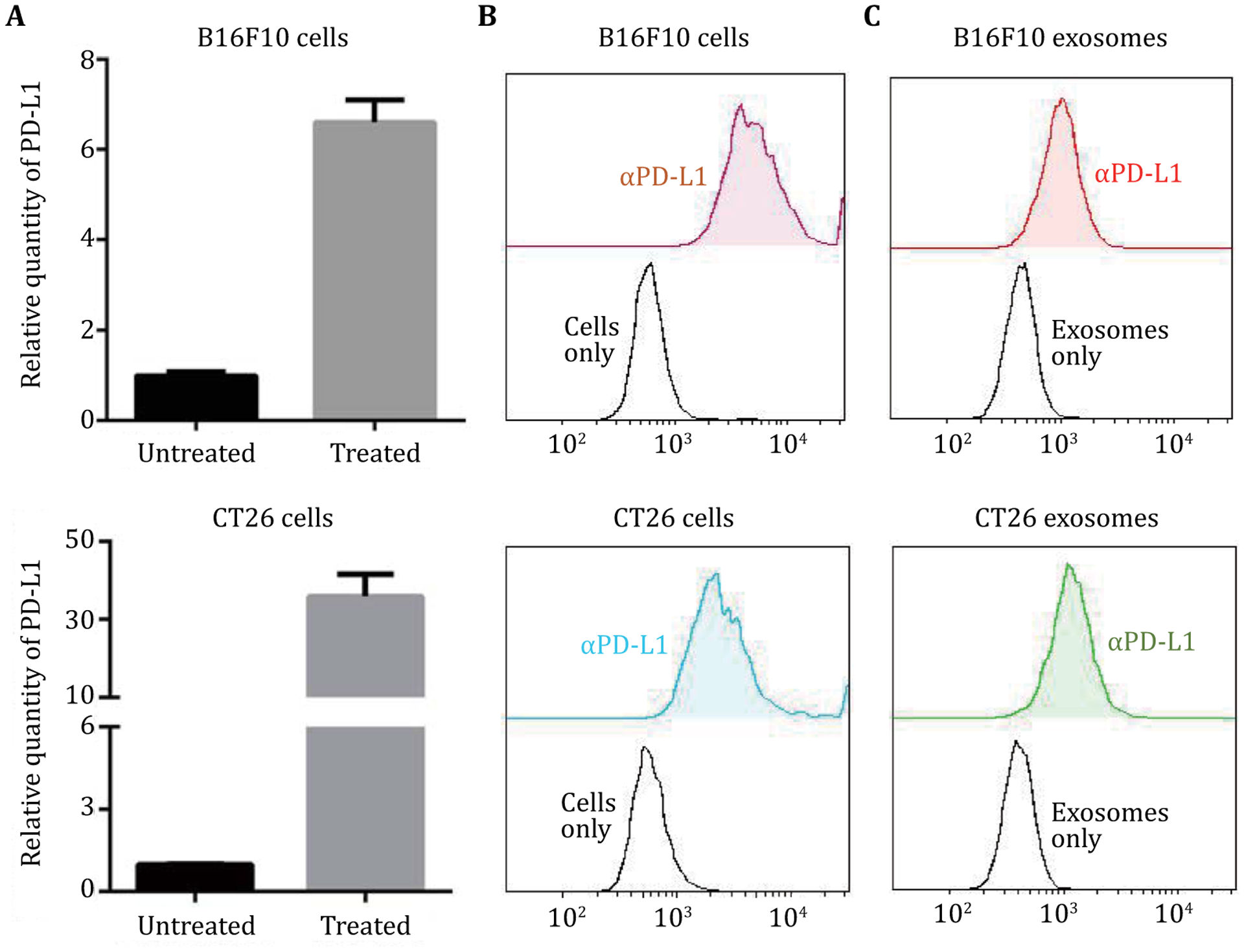
Generation of PD-L1-positive exosomes by immunostimulation of B16F10 melanoma cells and CT26 colorectal cancer cells. **A** qPCR results of the relative PD-L1 mRNA levels in whole cells. Immunostimulation treatment: 100 ng/mL mouse IFN-γ for 48 h. Data represent mean ± SD of three independent experiments. **B**, **C** Flow cytometry results verified the upregulated PD-L1 expression on the surface of immunostimulated B16F10 and CT26 cells (**B**) and on the corresponding exosomes (**C**). Samples were stained with αPD-L1-AlexaFluor647

We chose B16F10 mouse melanoma cells and CT26 mouse colorectal cancer cells as models, the corresponding tumors of which are poorly immunogenic and have an overall poor therapeutic response to ICIs (Chen *et al*. 2018; Lai *et al*. 2016). Prognostics that reveal the levels of immune checkpoints (*e.g*., PD-L1) would be highly valuable for patient stratification in personalized treatment. The steady-state PD-L1 expression level is low. Therefore, to generate exosomes that have high PDL1 expression levels as models for this study, B16F10 cells and CT26 cells were treated with recombinant mouse IFN-γ for immunostimulation that can eventually upregulate PD-L1 expression. After IFN-γ treatment for 48 h, the PD-L1 expression levels in these cells were quantified using qPCR. As shown in Fig. 2A, when cells were treated with 100 μg/mL of recombinant mouse IFN-γ, the PD-L1 expression levels increased by 5.60 ± 0.29 folds in B16F10 cells and 34.85 ± 3.36 folds in CT26 cells.

Technologies that isolate exosomes from biological fluids would be highly valuable for biological research and medical applications, including proteomic profiling (*e.g*., PD-L1) on exosomes. Ultracentrifugation has been commonly used for exosome isolation, which is however tedious and time-consuming, and requires advanced equipment and expertise (Théry *et al*. 2006). Precipitation-based methods, which use reagents such as polyethylene glycols (PEGs) followed by low-speed centrifugation, have also been used for exosome isolation (Weng *et al*. 2016). However, both ultracentrifugation- and precipitation- based methods can cause the fusion of the particles with contaminants such as proteins, thereby changing the biophysical properties of the exosomes and the sensitivity of exosome proteomic analysis (van der Pol *et al*. 2012; Rood *et al*. 2010; Linares *et al*. 2015). In this study, we implemented a fast affinity-based method for magnetic isolation of exosomes, using magnetic beads that were coupled with αCD63 antibody, using biotinylated αCD63 and streptavidin-coated magnetic beads. CD63 is a common marker protein of exosomes from a variety of tumor cells, including B16F10 cells and CT26 cells (Chen *et al*. 2016, 2018; Logozzi *et al*. 2009). This method allows recovery of intact exosomes from cell culture media for high throughput applications.

The PD-L1 expression on exosome surfaces was validated by the binding of PD-L1 with αPD-L1. Specifically, using mouse AlexaFluo647-labeled αPD-L1 by flow cytometry (Fig. 2B, C), we verified the binding ability of αPD-L1 with recombinant PD-L1 coupled on sepharose microbeads (supplementary Fig. S1), PD-L1 on the surface of IFN-γ-treated B16F10 cells and CT26 cells (Fig. 2B), and PD-L1 on the surfaces of exosomes derived from IFN-γ-treated cells (Fig. 2C), respectively.

### Design and characterization of HCR

**Fig. 3:**
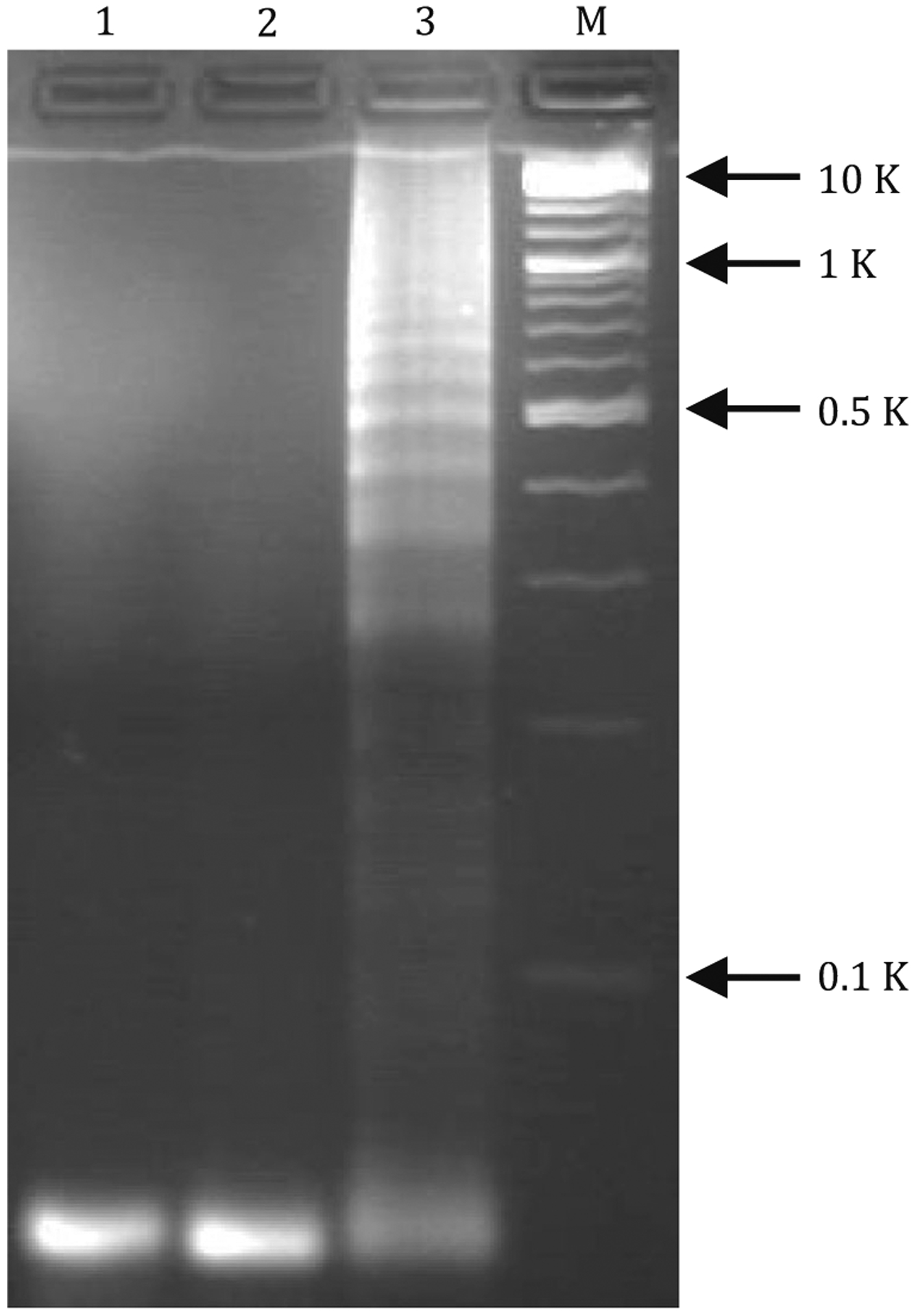
An agarose gel electrophoresis image verified the production of long DNA structures by HCR. Reaction condition: 5× SSCT buffer (5× SSC with 0.1% Tween 20) at room temperature for 1.5 h. Lane 1: H1; Lane 2: H2; Lane 3: T, H1, H2; M: Marker

The PD-L1 level on tumor cell-derived exosomes is positively correlated to the therapeutic response of ICB, which provides the basis for PD-L1 profiling in patient stratification via minimally invasive liquid biopsy. To overcome the generally low PD-L1 expression level on small exosomes relative to cells, we attempted to amplify the fluorescence signal intensity during PD-L1 profiling via *in situ* HCR. After PD-L1 expression was validated on exosomes, we then harnessed αPD-L1 as a ligand for signal amplification in PD-L1-specific ExoHCR. Specifically, we again used αCD63-coupled magnetic beads to isolate exosomes for *in-situ* HCR on PD-L1-positive exosome surfaces (Fig. 1). HCR was designed to assemble long repetitive DNA polymers from three DNA strands: a DNA initiator (T) and two hairpin monomers (H1 and H2) (Choi *et al*. 2014). In the absence of T, metastable H1 and H2 monomers remain to be monomers. In the presence of T or αPD-L1-T, T initiates a cascade of hybridization between H1 and H2 via base-pairing as characterized with the following steps in each cycle: (1) T (corresponding to DNA segments *ab* in Fig. 1) hybridized with the single-stranded toehold (DNA segment *a*′ of H1 and then with DNA segment *b*′), which results in the upfolding of H1 hairpin structure to expose the other half of H1 (DNA segments *bc*′); (2) similarly, exposed *bc*′ hybridized with DNA segments *b*′*c* in H2 and opened up the DNA fragment *ab* in H2; and (3) fragment *ab* in H2 has the identical sequence as DNA T and initiate another cycle of H1/H2 hybridization as in Step (1). As a result, a long dsDNA structure was generated on one T initiator with multiple copies of H1 and H2 via a chain reaction of alternating H1 and H2 polymerization. When H1 and H2 were labeled with a fluorophore, HCR provides a platform for *in situ* signal amplification. As shown in agarose gel electrophoresis, HCR was verified by the generation of long DNA strands using a molar ratio of T:H1:H2 at 1:10:10, using 5× SSCT buffer (5× SSC with 0.1% Tween 20) for 1.5 h at room temperature (Fig. 3). In order to conduct PD-L1-specific HCR by using αPD-L1, we synthesized αPD-L1-DNA conjugates in which one copy of αPD-L1 can be conjugated with multiple copies of T as HCR initiator. Note that the presence of multiple DNA T conjugated with one copy of αPD-L1 would enable multiple long dsDNA HCR products on one αPD-L1, which provides an additional mechanism of signal amplification in PD-L1 detection.

### PD-L1-specific ExoHCR for sensitive PD-L1 detection on tumor cell exosomes

**Fig. 4:**
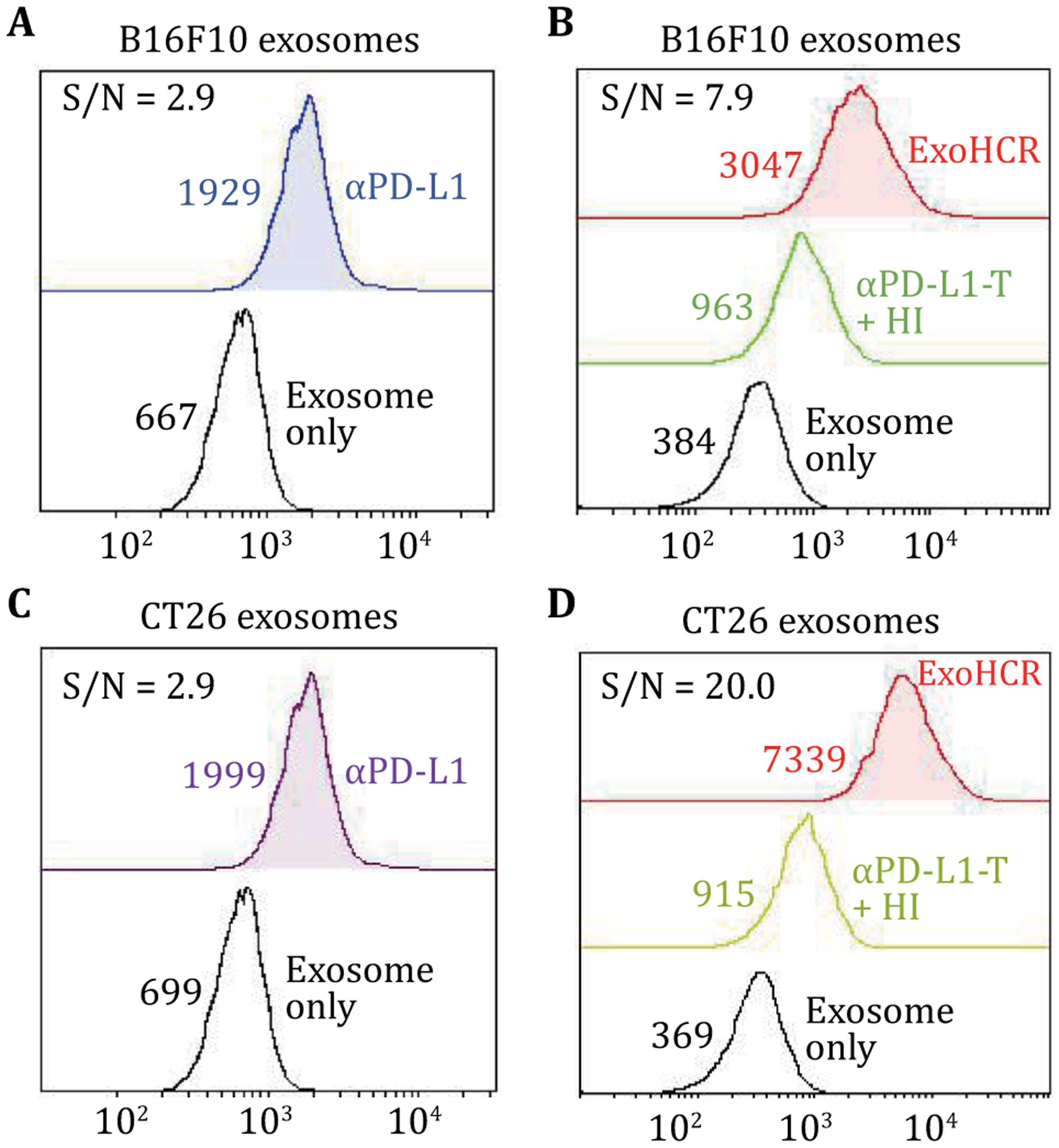
Flow cytometry results of exosomes which showed that ExoHCR enabled fluorescence signal amplification for PD-L1 profiling on cancer cell-derived exosomes. Compared with AlexaFluo647-labeled αPD-L1 (**A**, **C**), dye-labeled ExoHCR (**B**, **D**) significantly amplified the fluorescence intensities of exosomes derived from immunostimulated B16F10 cells (**A**, **B**) and CT26 cells (**C**, **D**). Values are the mean fluorescence intensities (MFIs). MFIs and signal-to-background (S/N) ratios of exosomes were detected by αPD-L1 and ExoHCR from B16F10 cells and CT26 cells

ExoHCR not only ensures the specificity via the αPD-L1 antibody but also increases the sensitivity of detection using HCR. To estimate the fluorescence signal gain per HCR product, we compared the fluorescence signal produced by AlexaFluo647-labeled αPD-L1 to the signal produced using αPD-L1-based ExoHCR. Specifically, for B16F10-derived exosomes, the mean fluorescence intensity (MFI) of AlexaFluo647-labeled αPD-L1 increased by only 2.7-fold relative to the background (unstained exosomes), while the MFI of ExoHCR increased by 6.9-fold (Fig. 4A, B), resulting in a 5.0-fold enhancement of the signal-to-noise ratios. For CT26-derived exosomes, the MFI of AlexaFluo647-labeled αPD-L1 increased by 3.3-fold, while the MFI of ExoHCR increased by 5.3-fold relative to the background (Fig. 4C, D), representing a 17.1-fold enhancement of the signal-to-noise ratios. Immunohistochemistry (IHC) has been used for PD-L1 expression profiling on tumor tissues. However, IHC-based PD-L1 profiling requires to use tissue samples that are often invasively collected, and can have limited inaccuracy due to vast tumor heterogeneity including intercellular heterogeneity of PD-L1 expression in tumor tissues (Pinato *et al*. 2016; Liu *et al*. 2017). By contrast, ExoHCR uses blood samples that are collected by minimally invasive liquid biopsy, and the use of systemic blood rules out potential inaccuracy caused by tumor heterogeneity. These features enable ExoHCR with great potential for PD-L1 profiling.

**Fig. 5:**
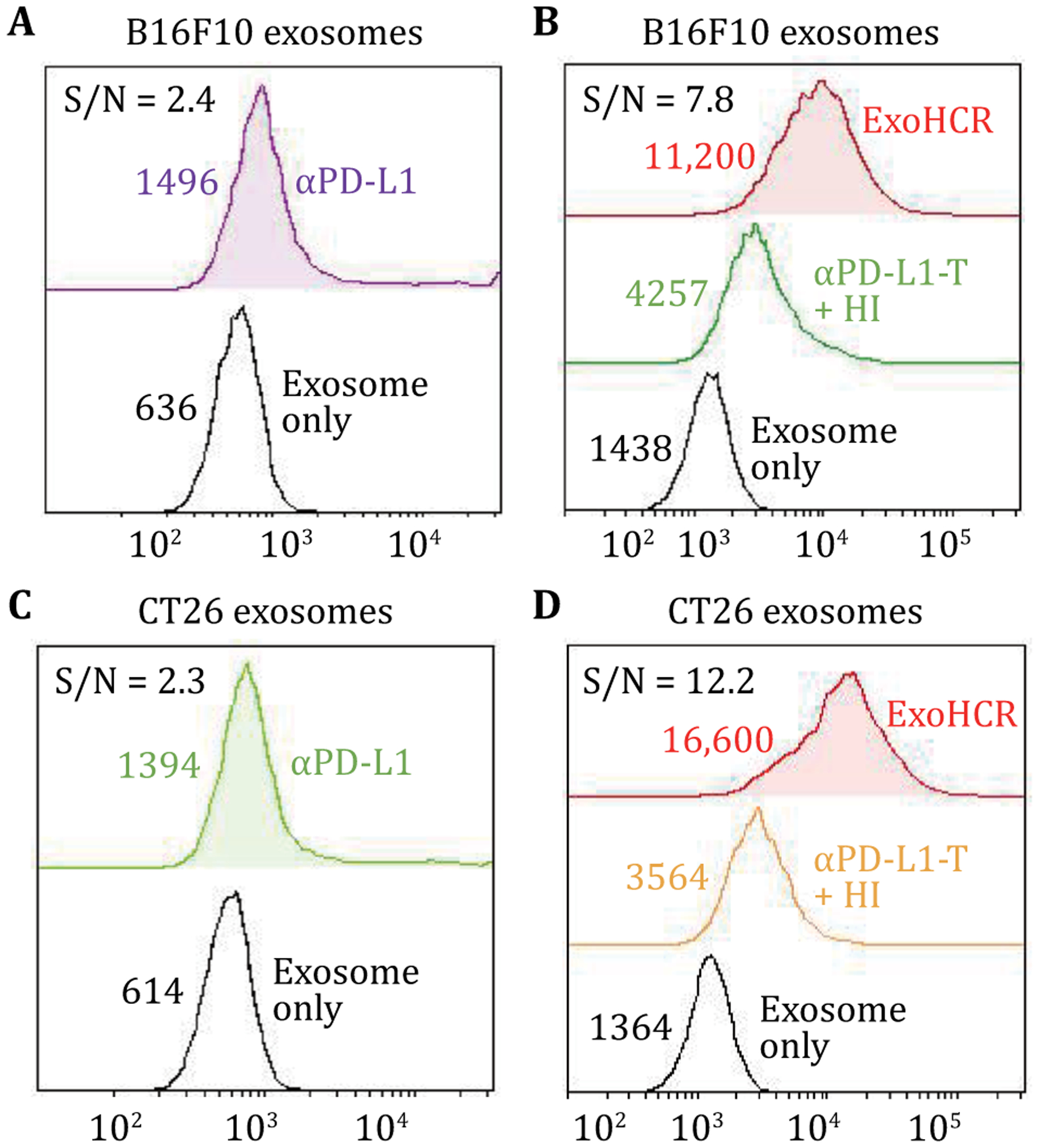
Flow cytometry results of exosomes which showed that ExoHCR enabled fluorescence signal amplification for PD-L1 profiling on exosomes from B16F10 cells (**B**) and CT26 cells (**D**) supernatant with 50% FBS. AlexaFluo647-labeled αPD-L1 was used as a control for PD-L1 analysis on B16F10-derived exosomes (**A**) and CT26-derived exosomes (**C**). Values are the MFIs

Furthermore, to evaluate the biostability of ExoHCR for future application using blood samples, we studied ExoHCR for PD-L1 detection in a blood-mimicking buffer supplemented with 50% FBS. As shown in Fig. 5A and B, for B16F10-derived exosomes, the signal-to-noise ratio of αPD-L1-based ExoHCR resulted in about 5.4-fold enhancement relative to AlexaFluo647-labeled αPD-L1. For CT26-derived exosomes (Fig. 5C, D), the signal-to-noise ratio of αPD-L1-based ExoHCR showed about 9.9-fold enhancement relative to AlexaFluo647-labeled αPD-L1.

## CONCLUSIONS

Prognostics that predict the cancer immunotherapeutic responses to ICB would be highly valuable for patient stratification and for optimal treatment design. The expression levels of immune checkpoints, such as PD-L1, are associated or even correlated with the corresponding ICIs, making these immune checkpoints promising prognostic biomarkers. Current standard-of-care practice of immune checkpoint profiling largely rely on IHC, which is intrinsic invasive and limited by complications such as tumor heterogeneity. To address these challenges, herein, we developed ExoHCR as a platform to interrogate immune checkpoint levels using systemic blood samples that can be collected by minimally invasive liquid biopsy. We demonstrated this principle using PD-L1 as a model. Recent studies have demonstrated that PD-L1 expression levels on tumor cell-derived exosomes are positively correlated with the therapeutic responses of anti-PD-L1 ICIs. Based on this, we induced PD-L1 expression on the exosomes of two poorly immunogenic tumor models, B16F10 melanoma and CT26 colorectal cancer, the ICB therapeutic responses are typically poor and are correlated with tumor PD-L1 expression levels. ExoHCR has several notable features: (1) ExoHCR uses blood samples that can be collected from patients with minimal invasiveness; (2) ExoHCR can bypass any inaccuracy caused by tumor heterogeneity by using tumor-derived exosomes from systemic blood; (3) ExoHCR uses two mechanisms for signal amplifications in sensitive target detection: multiple copies of HCR products were synthesized on one antibody, and multiple copies of H1/H2 monomers are tethered onto each HCR initiator (T); (4) the entire procedure of ExoHCR is simple, fast (a total of ~2 h), enzyme-free, and isothermal; (5) the immune-checkpoint-binding ligand (*i.e*., αPD-L1 in this study as a model) grants the specificity of PD-L1 detection; (6) this platform of ExoHCR can be widely applicable, because it can be readily adapted for the exosomal profiling of human immune checkpoints or other surface immune checkpoints by simply changing the antibody. Taken together, we envision that ExoHCR can be a non-invasive, simple, fast, sensitive, and broadly applicable prognostic platform for the profiling of exosomal biomarkers, including immune checkpoints for the prediction of ICB therapeutic responses in patient stratification.

## MATERIALS AND METHODS

### Materials and apparatus

Recombinant mouse IFN-γ was purchased from Pepro-Tech. Alexa Fluor 647 C2-maleimide was purchased from Invitrogen. Alexa Fluor 647 C2-maleimide was dissolved in dimethyl sulfoxide (DMSO) at a concentration of 6.7 mmol/L. Mouse αPD-L1 antibody was purchased from Bio X Cell. Sulfo-EMCS was purchased from bioWORLD. The streptavidin magnetic beads (PureProteome) were purchased from Millipore. Biotinylated mouse αCD63 antibody was purchased from Novus Biologicals. RNase-free DNase I was purchased from Lucigen Corporation. UltraPure 20× SSC buffer was purchased from Invitrogen. Tween 20 was purchased from Fisher Scientific. NAP-5 and PD-10 columns were purchased from GE Healthcare. Biotinylated mouse PD-L1 protein was purchased from ACROBiosystems.

### Cell culture

B16F10 mouse melanoma cells and CT26 mouse colorectal cancer cells were purchased from ATCC. B16F10 cells were cultured in Dulbecco’s Modified Eagle’s Medium (DMEM) (Sigma) supplemented with 10% (*v*/*v*) fetal bovine serum (FBS) (Corning), 100 U/mL penicillin, and 100 μg/mL streptomycin. CT26 cells were cultured in RPMI-1640 medium (Sigma) supplemented with 10% (*v*/*v*) FBS, 100 U/mL penicillin, and 100 μg/mL streptomycin. For immunostimulation with IFN-γ, cells were incubated with 100 ng/mL of recombinant mouse IFN-γ for 48 h (Chen *et al*. 2018).

### Quantitative PCR (qPCR)

Total RNA was isolated from B16F10 and CT26 cells using TRIzol Reagent (Invitrogen), and reverse transcribed into first-strand complementary DNA (cDNA) with random primer with RevertAid First-Strand cDNA Synthesis Kit (ThermoFisher Scientific). The samples were then analyzed by qPCR using the Power SYBR Green PCR master mix (Thermo Fisher Scientific) in an Applied Biosystems QuantStudio 3 Real-Time PCR system. All qPCR amplifications were carried out in 20 μL volume in triplicate. qPCR was performed by the ΔΔ*C*_T_ method (Livak and Schmittgen 2001). GAPDH was used as a reference. All primers were ordered from Integrated DNA Technologies (IDT) (see sequences in supplementary Table S1).

### Preparation of αPD-L1-AlexaFluor647 conjugate

After being degassed for 10 min, αPD-L1 was incubated with Alexa Fluor 647 C2-maleimide (Invitrogen) in PBS (pH 7.4) overnight at 4°C in the dark, and stirred by a magnetic stirring bar. The unreacted Alexa Fluor 647 C2-maleimide was removed by Diol200 gel filtration column chromatography (Kawabata *et al*. 2005). αPD-L1-AlexaFluor647 was collected and stored at 4 °C for future use.

### αPD-L1 binding assays

#### αPD-L1 binding with cells

Cells were pretreated with IFN-γ (100 ng/mL) in the culture medium for 48 h before use (Chen *et al*. 2018). Cells were collected and washed with Dulbecco’s Phosphate Buffered Saline (DPBS). αPD-L1-AlexaFluor647 (5 μg/mL) was then added to 200 μL cell suspension with a density of 5 × 10^6^ cells/mL and incubated with gentle agitation on ice for 30 min in the dark. Then, cells were washed with DPBS for three times, collected after centrifugation at 1000 r/min for 3 min, and resuspended in 600 μL of DPBS. Finally, the cells were assayed by a Canto II flow cytometer (BD Bioscience, CA). Ten thousand cells were acquired and analyzed with FlowJo software.

#### αPD-L1 binding with cell-derived exosomes

First, 15 μL biotinylated mouse αCD63 antibody was mixed with streptavidin-coated magnetic beads and incubated with gentle agitation for 30 min at room temperature. The resulting αCD63-conjugated beads were then collected using a magnet and washed three times. For immunostimulation to enhance PD-L1 expression, B16F10 or CT26 cells were treated with IFN-γ (100 ng/mL) for 48 h. Cell culture medium was then harvested and incubated with αCD63-conjugated beads, followed by incubation with gentle agitation for 30 min at room temperature. The αCD63-bead-bound exosomes were harvested using a magnet again and washed three times. Then, the αPD-L1-AlexaFluor647 (5 μg/mL) was added to the above αCD63-bead-bound exosomes, followed by incubation with gentle agitation for 30 min at room temperature in the dark. Finally, exosomes were washed by magnetic collection, resuspended, and assayed by flow cytometry on a Canto II flow cytometer (BD Bioscience, CA). Ten thousand beads were acquired and analyzed with FlowJo software.

#### αPD-L1 binding with mouse PD-L1 protein

Firstly, biotinylated mouse PD-L1 protein was mixed with 100 μL streptavidin-coated sepharose beads and incubated with gentle agitation for 30 min at room temperature. The resulting beads were subsequently harvested by centrifugation and washed for three times. Next, the αPD-L1-AlexaFluor647 (5 μg/mL) was added to PD-L1-coated sepharose beads and incubated with gentle agitation for 30 min at room temperature in the dark. Then, beads were washed, collected, resuspended, and assayed by flow cytometry on a Canto II flow cytometer (BD Bioscience, CA). Ten thousand beads were acquired and analyzed with FlowJo software.

### DNA design for HCR

The trigger DNA strand (T) is 36 nucleotide (nt) long, and HCR hairpins (H1 and H2) are 72 nt long (12-nt toehold, 24-bp stem, 12-nt loop) (see sequences in supplementary Table S2) (Choi *et al*. 2014). To ensure that H1 and H2 form hairpin monomers, the hairpin DNA strands were snap-cooled in 5× SSC buffer before use (heat at 95 °C for 90 s, cool to room temperature for 30 min).

### HCR and agarose gel electrophoresis

H1 and H2 were snap-cooled separately at 3 μmol/L in 5× SSC buffer. T was diluted to 0.3 μmol/L in DNase-free ultrapure water. HCR was performed in 5× SSC with 0.1% Tween 20 at room temperature for 1.5 h (Choi *et al*. 2014). HCR was verified by agarose gel electrophoresis, in which each lane was prepared by mixing 20 μL 5× SSC, 3 μL 10× SSC with 1% Tween 20, 1 μL ultrapure water, 2 μL the trigger and 2 μL of each hairpin. In the absence of T, 2 μL ultrapure water as a substitution was added to make the total reaction volume to 30 μL. The samples were supplemented with DNA gel loading dye (6×) and loaded into a native 2% agarose gel containing final concentration of 0.5 μg/mL of ethidium bromide (EB). Gel electrophoresis was run in 1× TAE buffer at 120 V for 40 min at room temperature, before imaging in a BioRad Gel Doc imager.

### Synthesis of mouse αPD-L1-DNA conjugate

Amine-functionalized trigger DNA (T) (1 μmol/L) was mixed with Sulfo-EMCS (50 μmol/L) in PBS (pH 7.4) at room temperature for 2 h. Next, the resulting solution was desalted using NAP-5 Desalting Column (GE Healthcare) to purify maleimide-activated DNA T (Griepenburg *et al*. 2013). Then, 20 μL αPD-L1 was added to maleimide-activated DNA T. After incubation for 1 h at room temperature, mouse αPD-L1-DNA was purified using a PD-10 column (GE Healthcare) (Enomoto *et al*. 2008).

### *In situ* ExoHCR on exosomes exosomal PD-L1 profiling by flow cytometry

From culture medium of B16F10 and CT26 cells that had been treated with 100 ng/mL of recombinant mouse IFN-γ for 48 h, exosomes were harvested from B16F10 and CT26 cell culture medium (300 μL) using αCD63-conjugated magnetic beads (5 μL) as described above. The resulting exosomes were then mixed and incubated with mouse αPD-L1-T conjugate (5 μg/mL) at room temperature for 30 min. Exosome solution was washed using a magnet to remove excessive antibody-DNA conjugates. Next, H1 and H2 (3 μmol/L) were added to the resulting exosome solution, followed by incubation in 5× SSC with 0.1% Tween 20 at room temperature for 1.5 h in the dark. Finally, exosomes were washed, collected, resuspended, and assayed by flow cytometry on a Canto II flow cytometer (BD Bioscience, CA). Ten thousand beads were acquired and analyzed using FlowJo software. The signal-to-background ratio was designated to estimate the fluorescence signal gain. αPD-L1-AlexaFluor647 was used as a control. Moreover, to investigate the biostability of ExoHCR, we repeated the above study by spiking exosomes in cell culture medium supplemented with 50% FBS in order to mimic exosomes harvested from blood samples.

### Statistical analysis

Data were expressed as means ± standard deviation (SD). The statistical analyses of differences between two groups were analyzed using Student’s *t*-test. The analyses were performed with SPSS version 20.0, and statistical significance was accepted at least at the 1% level.

## Supplementary Material

Zhu Supplement
