# Supplementary material for: ExoHCR: a sensitive assay to profile PD-L1 level on tumor exosomes for immunotherapeutic prognosis
Source: Biophys Rep. Author manuscript; Available in PMC 2021 Jul 29. (PMC8320673; doi:10.1007/s41048-020-00122-x)
Supplement: Zhu Supplement [file NIHMS1672984-supplement-Zhu_Supplement.pdf]

Supplementary materials

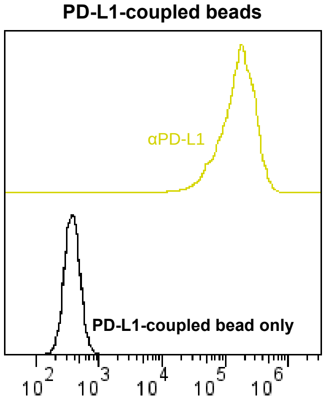

Fig. S1 Flow cytometry results verified the binding of αPD-L1 to PD-L1. Incubation of αPD-L1-AlexaFluor647 with PD-L1 protein coupled on sepharose beads led to fluorescence enhancement as shown in flow cytometry analysis

Table S1 Primer sequences used in qPCR

Primers	Sequences (5'-3')
mPD-L1 For (Casey *et al.* 2016)	GCTCCAAAGGACTTGTACGTG
mPD-L1 Rev	TGATCTGAAGGGCAGCATTTC
GAPDH For	AGGGAAGAGGCCAAGATTAAAG
GAPDH Rev	CTGCTGAGACCAGAAGAATCC

Table S2 DNA sequences used in HCR

Name	Sequence (5'-3')
T	Amino-TTTTTTTTTTAGCTCAGTCCATCCTCGTAAATCCTCATCAATCATC
H1	FAM-CCTCGTAAATCCTCATCAATCATCCAGTAAACCGCCGATGATTGATGAGGATTTACGAGGATGGACTGAGCT
H2	GGCGGTTTACTGGATGATTGATgAGGATTTACGAGGAGCTCAGTCCATCCTCGTAAATCCTCATCAATCATC-FAM
